# Vade Mecum of Ophthalmological Therapeutics

**Published:** 1898-06

**Authors:** 


### Book Reviews.

Vade Mecum of Ophthalmological Therapeutics. By Dr. Landolt and Dr. Gygax. Philadelphia: J. B. Lippincott Company. 1898.

In the introduction of this concise, though excellent little work, the authors state that it is not a treatise nor a dictionary. They aim to give a constant companion to the busy practitioner or student preparing for examination. They presuppose everyone can diagnosticate the various ocular diseases, otherwise the volume would be of no practical value. There are 326 numbered paragraphs, arranged in alphabetical order of the various eye diseases, also the appropriate treatment, systemic and local. The pharmaceutical part has evidently been prepared with care and includes drugs most successfully used for the diseases described.

The direction under antiseptics for cataract operation—to cut off eye lashes and eyebrows—would hardly be popular in this country and seems unnecessary. The introduction of the sulphate of atropia and the sulphate of eserin in the pure state between the lids has not been tried much in this country among the leading ophthalmologists, as the danger of absorption into the circulation must be vastly increased by this method.

Under muscular insufficiencies Dr. Landolt brings forward his well-known ideas of advancement of the weaker in preference to tenotomy of the stronger muscle. Opinions in this country still differ greatly on this point. "The patient hardly ever tolerates permanently the glass completely correcting his myopia" is timely and can be verified by the experience of many who have tried prescribing a full correction. R. H. S.

